# Waldenström Macroglobulinemia Diagnosed by Ultrasonography‐Guided Biopsy of the Right Perinephric Tumor

**DOI:** 10.1002/iju5.70022

**Published:** 2025-04-09

**Authors:** Shingo Morinaga, Shigeyuki Aoki, Motoi Tobiume, Genya Nishikawa, Fusako Higuchi, Yuusuke Ikenohata, Manabu Honda, Hiroe Kubo, Tomoko Sawada, Yoshiaki Yamada

**Affiliations:** ^1^ Department of Urology Japan Community Health Care Organization Kani Tono Hospital Kani Gifu Japan; ^2^ Department of Urology Japan Organization of Occupational Health and Safety Asahi Rosai Hospital Owariasahi Aichi Japan; ^3^ Department of Clinical Laboratory Japan Community Health Care Organization Kani Tono Hospital Kani Gifu Japan; ^4^ Division of Nursing Japan Community Health Care Organization Kani Tono Hospital Kani Gifu Japan; ^5^ Division of Radiology Japan Community Health Care Organization Kani Tono Hospital Kani Gifu Japan; ^6^ Division of Hospital and Clinic Coordination Japan Community Health Care Organization Kani Tono Hospital Kani Gifu Japan

**Keywords:** lymphoplasmacytic lymphoma, malignant lymphoma, perinephric tumor, ultrasonography‐guided biopsy, Waldenström macroglobulinemia

## Abstract

**Introduction:**

Waldenström macroglobulinemia is a low‐grade B‐cell lymphoma characterized by lymphoplasmacytic lymphoma infiltration of the bone marrow and immunoglobulin M (IgM) protein.

**Case Presentation:**

An 80s‐year‐old male presented to our hospital with chief complaints of weight loss and general fatigue. Computed tomography (CT) showed homogeneous tumor around the kidney with elevated soluble Interleukin‐2 receptor, serum IgM, and β2‐microglobulin levels. Histopathological analysis by ultrasonography‐guided biopsy revealed dense lymphocytic proliferation, plasmacytoid differentiation, and Dutcher bodies, positive for CD20, CD138, and IgM, but negative for CD3 and CD5, consistent with lymphoplasmacytic lymphoma. Bone marrow biopsy revealed infiltration of the lymphoplasmacytic lymphoma. The patient received four courses of bortezomib, cyclophosphamide, and dexamethasone along with dexamethasone, rituximab, and cyclophosphamide therapy. Twelve months after treatment, CT revealed only slightly enlarged abdominal para‐aortic lymph nodes.

**Conclusion:**

Malignant lymphoma in perinephric lesions is a relatively rare condition; however, a definitive diagnosis can be obtained by ultrasound‐guided biopsy, allowing early initiation.

**Abbreviations**
β2‐MG: β2‐microglobulin
Alb: albumin
cCR: clinical complete response
CRN: creatinine
CT: computed tomography
DRC: dexamethasone, rituximab and cyclophosphamide
Hb: hemoglobin
IgM: immunoglobulin M
LDH: lactate dehydrogenase
LPL: lymphoplasmacytic lymphoma
ML: malignant lymphoma
*MyD88*
: myeloid differentiation factor 88
Plt: platelet
PS: performance status
PSA: prostate specific antigen
sIL‐2R: soluble Interleukin‐2 Receptor
VCD: bortezomib, cyclophosphamide and dexamethasone
WM: Waldenström macroglobulinemia

**Summary**

- Malignant lymphoma occurring in the perinephric lesions is extremely rare.
- We describe a case of Waldenström macroglobulinemia where right perinephric malignant lymphoma was suspected based on laboratory examinations and computed tomography findings, diagnosed by ultrasound‐guided biopsy, bone marrow biopsy, and immunoglobulin M protein.

## 1 Introduction

WM is an LPL in which lymphoplasmacytic cells, differentiating from B lymphocytes into plasma cells, lead to tumor growth, with bone marrow involvement and excessive production of monoclonal IgM, accounting for 90%–95% of LPLs [1]. It is rare, comprising 2% of all hematologic tumors [2], and was first described by Waldenström in 1944 [3].

## 2 Case Presentation

An 80‐year‐old man presented to the Department of General Internal Medicine of our hospital with the chief complaints of a 3‐month weight loss history of approximately 3 kg/month and general fatigue. Laboratory examination revealed anemia, and abdominal CT revealed a mass resembling a subcapsular renal hematoma around the right kidney, prompting a referral to the Department of Urology. There was no history of smoking or drinking, and no notable history about his family His past medical history included colonoscopic polypectomy and ongoing oral medication for hypertension.

On admission, his height was 164 cm, weight was 55 kg, he was conscious, had a body temperature of 36.1°C, blood pressure of 145/84 mmHg, pulse rate of 55/min (regular), there was pallor in the palpebral conjunctiva, with a flat and soft abdomen, and no hepatosplenomegaly. His PS was 0 with no signs of subcutaneous bleeding due to falls or bruising of the back.

Laboratory examination showed Hb 9.1 g/dL, Plt 209 000/μL, Alb 2.7 g/dL, LDH 136 U/L, CRE 2.63 mg/dL, IgM 7.705 mg/dL, and β2‐MG 6.3 mg/L. Anemia, hypoalbuminemia, renal dysfunction, and elevated IgM and β2‐MG levels were observed. Tumor markers showed normal PSA and elevated sIL‐2R levels (1016 U/mL). Urinalysis and urine cytology revealed no abnormalities.

**FIGURE 1:**
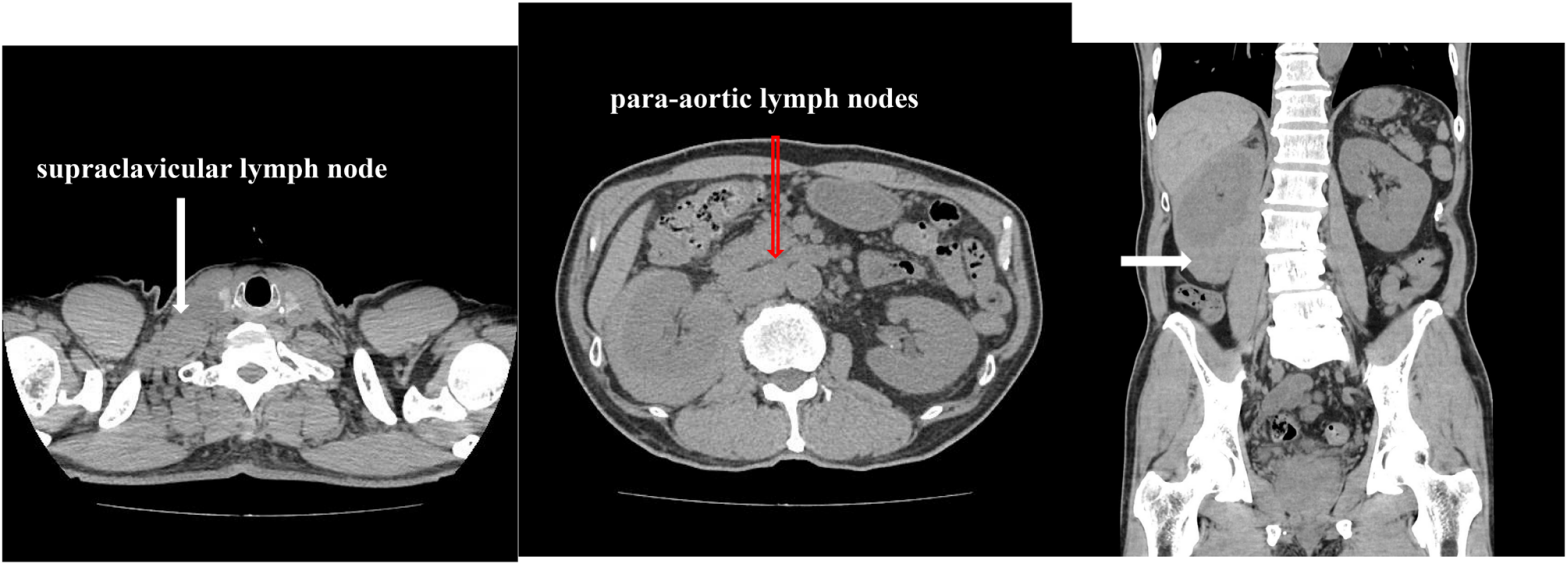
Chest plain computed tomography(CT) did not reveal any abnormal findings in the lung field but did reveal an enlarged right supraclavicular lymph node (⇩). Plain abdominal CT revealed a tumorous lesion around the right kidney. The tumor had a clear border and was homogeneous inside, with the same density as the renal parenchyma (⇨). In addition, enlarged para‐aortic lymph nodes were also observed (⇩).

**FIGURE 2:**
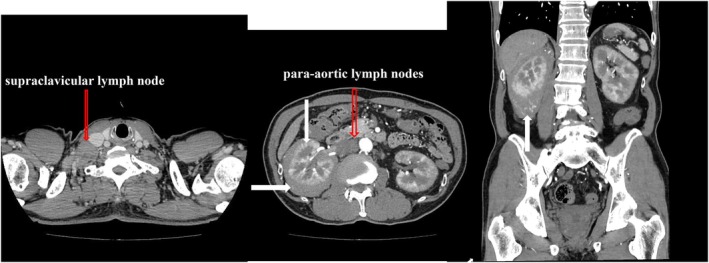
Contrast‐enhanced computed tomography showed that the perinephric tumor was weakly enhanced compared to the renal parenchyma (⇨). The morphology of the right kidney was maintained, and no obvious tumor infiltration into the renal parenchyma was observed (⇩). No necrotic or degenerative findings were observed within the tumor (⇨), and the vascular structure within the tumor was maintained (⇧).

Plain and contrast‐enhanced chest CT revealed no lung abnormalities; however, enlarged right supraclavicular lymph nodes were observed. Plain abdominal CT showed a perinephric tumor around the right kidney, with a clear homogeneous interior and the same density as the renal parenchyma (Figure 1). Contrast‐enhanced CT showed weak enhancement compared to the renal parenchyma, with no signs of infiltration or necrosis and preserved vascular structures. Enlarged abdominal para‐aortic lymph nodes were also observed (Figure 2).

**FIGURE 3:**
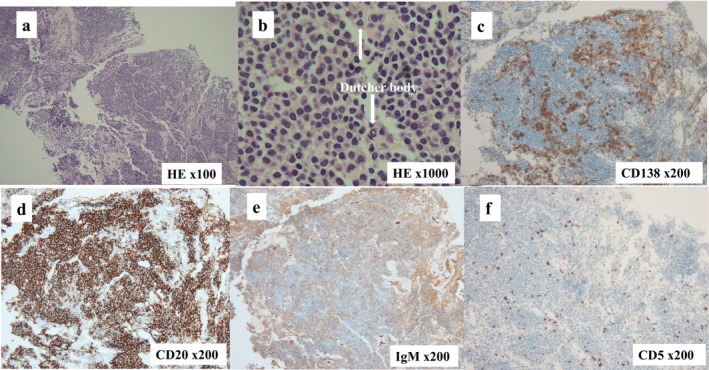
Histopathological analysis showed dense proliferation of atypical lymphocytes, plasmacytoid differentiation, and Dutcher bodies [4] (⇩) (a, b), positive for CD20, CD138, and IgM, and negative for CD3 and CD5 (c, d, e, f).

Based on laboratory examinations and imaging results, a tumor around the right kidney was suspected to be ML, and an ultrasound‐guided biopsy was performed. Histopathological analysis showed dense proliferation of atypical lymphocytes, plasmacytoid differentiation, and Dutcher bodies [4], positivity for CD20, CD138, and IgM, and negativity for CD3 and CD5, which was consistent with LPL (Figure 3). A bone marrow biopsy revealed LPL infiltration, with high IgM levels (7705 mg/dL) confirming the WM diagnosis.

**FIGURE 4:**
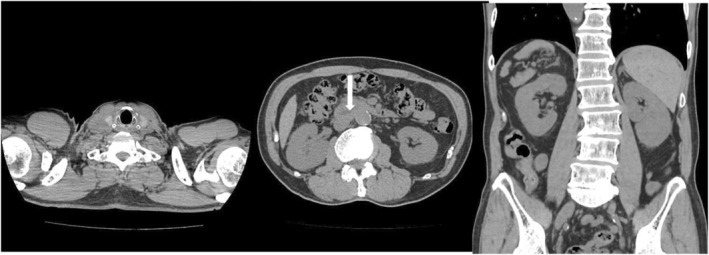
Twelve months after treatment, plain computed tomography shows that only slight enlargement of the abdominal para‐aortic lymph nodes is observed (⇩), and the right perinephric tumor and right supraclavicular lymph node continue to show clinical complete response.

In the Department of Hematology, the patient was treated with four courses of VCD therapy, and IgM decreased (2043 mg/dL); therefore, four courses of DRC therapy were administered as additionally. Twelve months after treatment, the tumor marker sIL‐2R levels returned to normal (321 U/mL), and renal function had improved to CRN 1.49 mg/dL. Plain CT showed only slight swelling of the abdominal para‐aortic lymph nodes, and the right perinephric tumor and right supraclavicular lymph nodes continued to show cCR (Figure 4).

## 3 Discussion

ML around the kidney is a relatively rare disease, comprising only 2.4%–14% of all extra‐nodal lymphomas [5]. WM, a subtype of ML, has an incidence rate of 2–3 cases/million annually [1], occurring more commonly among the elderly, with a reported male‐to‐female ratio of 1.4:1 [6]. Approximately 90% of patients have a somatic mutation in the *MyD88* gene, causing WM [7].

WM has few specific symptoms, but treatment is required for recurrent fever, weight loss, general fatigue, progressive lymphadenopathy, hepatosplenomegaly, anemia (Hb ≤ 10 g/dL) and thrombocytopenia (≤ 100 000/μL) due to bone marrow infiltration [8, 9, 10].

ML is definitively diagnosed by histopathological examination. The imaging findings of ML are as follows: (1) no necrotic tissue within the tumor compared to the tumor size; (2) a uniform low‐density tumor similar to the renal parenchyma on plain CT; (3) the contrast effect is lower density than that of the renal parenchyma on contrast‐enhanced CT, with preserved vascular structures within the tumor, growing compressing adjacent organs; and (4) lymph node enlargement around the aorta is observed in 66% of cases [11, 12, 13]. Our patient's imaging findings were consistent with the above characteristic imaging results, and suspecting ML, we performed an ultrasound‐guided biopsy for a definitive diagnosis. We believe that biopsy should be performed proactively when ML is suspected on imaging.

For WM treatment, which progresses slowly, treatment is not initiated if there are no symptoms, and it is indicated only if symptoms develop [9, 14]. Our patient also presented with weight loss, anemia, renal dysfunction, and general fatigue; therefore, treatment was necessary. Currently, there is no consensus on the optimal initial regimen for WM treatment, and it is usually considered on a case‐by‐case [1, 9]. It has been reported that patients with serum IgM levels > 4000 mg/dL are at risk of IgM flares; therefore, care is required when using rituximab [9]. Our patient received four courses of VCD therapy, which is recommended for the initial treatment of WM [15] were administered due to high IgM levels (7705 mg/dL). After IgM levels decreased to 2043 mg/dL, four courses of DRC therapy were administered as additional treatment.

The revised International Prognostic Scoring System for WM was reported in 2019 to predict WM [16]. According to this report, the score was determined based on age (≤ 65 vs. 66–75 vs. ≥ 76 years old are 0, 1, 2 points, respectively.), β2‐ MG ≥ 4 mg/L, LDH ≥ 250 IU/L, and serum albumin < 3.5 g/dL, with the 5‐year survival rate being 95% for score 0, 86% for score 1, 78% for score 2–3, and 36% for score 4–5. Our patient was 80 years old, serum albumin of 2.7 g/dL, and β2‐MG of 6.3 mg/L, giving a score of 4.

At first glance, WM appears to be a highly specialized disease that only hematologists diagnose and treat; however, its diverse clinical symptoms, imaging findings, and abnormal laboratory values, can be encountered by physicians across various departments. Therefore, it was considered understanding the disease characteristics is essential.

## 4 Conclusion

We reported a case of WM, a subtype of low‐grade B‐cell ML that occurred in a right perinephric lesion. ML occurring in the right perinephric lesion is extremely rare; however, suspected laboratory examination and imaging findings led to a definitive diagnosis via ultrasound‐guided biopsy, allowing early treatment.

## Consent

Written informed consent was obtained from the patient for publication of this case report and any accompanying images.

## Conflicts of Interest

The authors declare no conflicts of interest.
